# Clinical features of a rare anatomical variation of the posterior tibial and fibular arteries

**DOI:** 10.1590/1677-5449.003416

**Published:** 2016

**Authors:** Pedro Oliveira Portilho, Tulio Fabiano de Oliveira Leite, Ricardo Cardoso, Lucas Alves Sarmento Pires, Julio Guilherme Silva, Carlos Alberto Araujo Chagas

**Affiliations:** 1 Hospital da Força Aérea do Galeão, Rio de Janeiro, RJ, Brazil.; 2 Universidade Estadual Paulista – UNESP, São Paulo, SP, Brazil.; 3 Universidade Federal Fluminense – UFF, Niterói, RJ, Brazil.; 4 Universidade Federal do Rio de Janeiro – UFRJ, Rio de Janeiro, RJ, Brazil.

**Keywords:** anatomical variation, cadaver, fibular artery, posterior tibial artery, popliteal artery, variação anatômica, cadáver, artéria fibular, artéria tibial posterior, artéria poplítea

## Abstract

The posterior tibial artery normally arises from tibial-fibular trunk at the popliteal fossa, together with the fibular artery. The classic course of the posterior tibial artery is to run between the triceps surae muscle and muscles of the posterior compartment of the leg before continuing its course posteriorly to the medial malleolus, while the fibular artery runs through the lateral margin of the leg. Studies of both arteries are relevant to the fields of angiology, vascular surgery and plastic surgery. To the best of our knowledge, we report the first case of an anastomosis between the posterior tibial artery and the fibular artery in their distal course. The two arteries joined in an unusual “X” format, before division of the posterior tibial artery into plantar branches. We also provide a literature review of unusual variations and assess the clinical and embryological aspects of both arteries in order to contribute to further investigations regarding these vessels.

## Resumo

A artéria tibial posterior e a artéria fibular se originam do tronco tibiofibular, na fossa poplítea. A trajetória clássica da artéria tibial posterior é correr entre o tríceps sural e os músculos do compartimento posterior da perna, e, então, seguir posteriormente ao maléolo medial. Já a artéria fibular corre na margem lateral da perna, seguindo profundamente aos músculos. O estudo dessas artérias é relevante para o campo da angiologia, cirurgia vascular e cirurgia plástica. O presente trabalho é o primeiro relato de caso de uma anastomose entre ambas artérias, na porção distal de suas trajetórias. Tais artérias se anastomosaram em formato de “X”, antes da divisão da artéria tibial posterior em ramos plantares. Foi feita uma revisão de literatura das variações de tais artérias, dando ênfase ao aspecto clínico e embriológico, de modo a contribuir para novas investigações sobre esses vasos.

## INTRODUCTION

Many anatomical variations in the origins, trajectories, distribution and anastomoses of the arteries of the lower limbs have been described. The femoral artery supplies the thigh, while the popliteal artery supplies the leg and foot.^1^^-^ 7

The posterior tibial artery (PTA) and the fibular artery (FA) are branches of the tibial-fibular trunk (TFT), located at the popliteal fossa. The TFT usually describes a trajectory of 1 to 8 cm after the popliteal artery emits the anterior tibial artery (ATA). It is usually followed by two veins and the tibial nerve. The PTA runs down the posterior face of the leg, following the posterior surface of the tibia, accompanied by two veins, and is located posteriorly to the triceps surae muscle and anteriorly to the posterior tibialis muscle and the flexor digitorum longus muscle, giving off muscular branches for the posterior compartment.^3^^,^ 8 ^,^ 9

In the foot, the PTA runs along the medial retromaleolar canal, medially to the calcaneal tendon, emitting malleolar and calcaneal branches before dividing into the lateral and medial plantar arteries. It is responsible for the blood supply to the posterior muscles of the leg and the plantar region of the foot.^3^^,^ 8 ^,^ 9 At its origin, the FA runs laterally between the posterior tibialis muscle and the flexor hallucis muscle. It is also followed by two veins. The FA emits a nutrient artery for the fibula, muscular branches for the lateral and posterior muscles of the leg, and calcaneal branches.^3^^,^ 8 ^,^ 9

Variants differing from this pattern can be explained by segmental hypoplasia, abnormal fusions or absence.^4^^,^ 10 Knowledge of these variations is useful in the fields of angiology and vascular surgery, while in plastic surgery vascular flaps (either from the PTA or the FA) have been used to restore the contours and function of the mandible and other regions, meaning that the anatomy of these vessels is vital to the success of such procedures.^5^^,^ 11 ^-^ 13 Albeit uncommon, cases of pseudoaneurysms^14^ and aneurysms^15^ of the PTA have been reported in the literature.

Moreover, with the recent increase in the numbers of diabetic patients, critical limb ischemia due to multiple and large occlusions of the lower limb vessels is becoming more and more common; therefore knowledge of these arteries is needed in order to avoid amputations.^16^

We report a previously undescribed anatomic variation of the PTA and FA and present a review of significant anatomic variations and their clinical and embryological features.

## CASE REPORT

**Figure 1:**
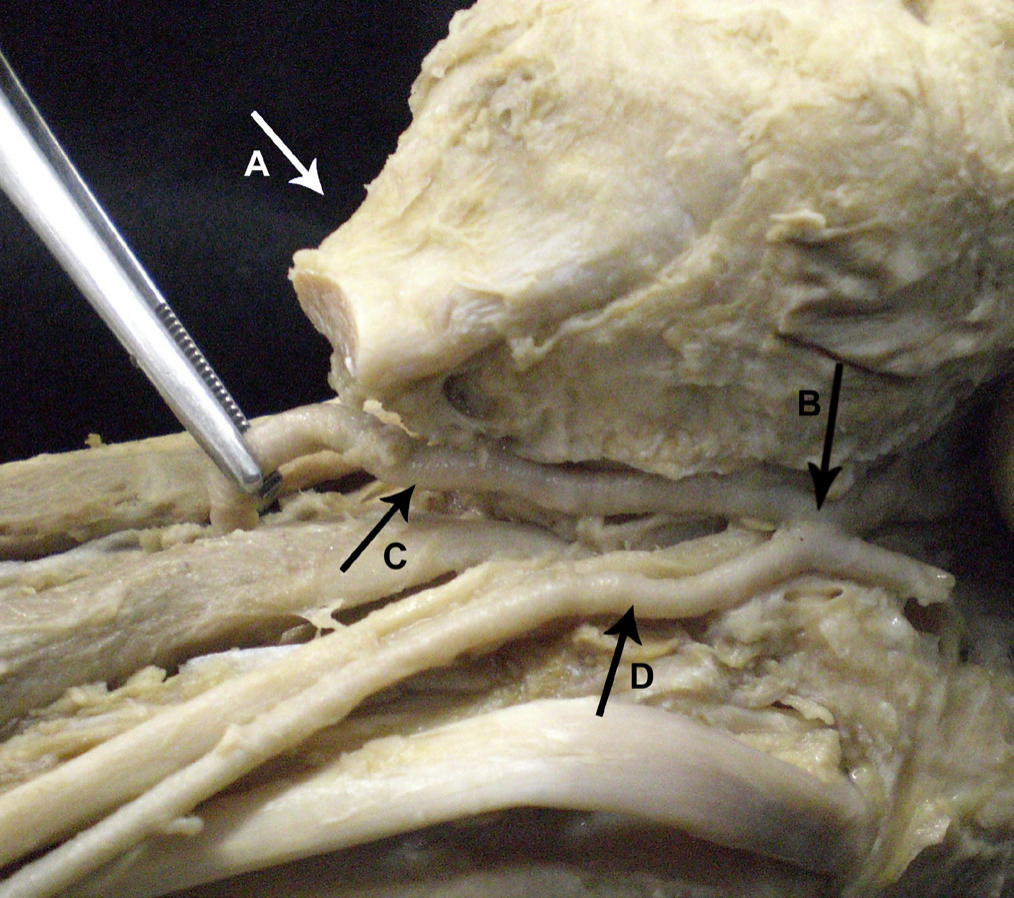
Medial view of posterior region of the right leg. The FA is visible (C). The triceps surae tendon (A), and the PTA (D) are seen following their regular course. The anastomosis is also visible (B).

A 50-year-old male cadaver fixed with a 10% formalin solution (cause of death unknown) was dissected during Anatomy classes. While dissecting the right lower limb, we observed an uncommon relationship between the PTA and the FA along their course (Figure 1). The left lower limb exhibited normal anatomy.

**Figure 2:**
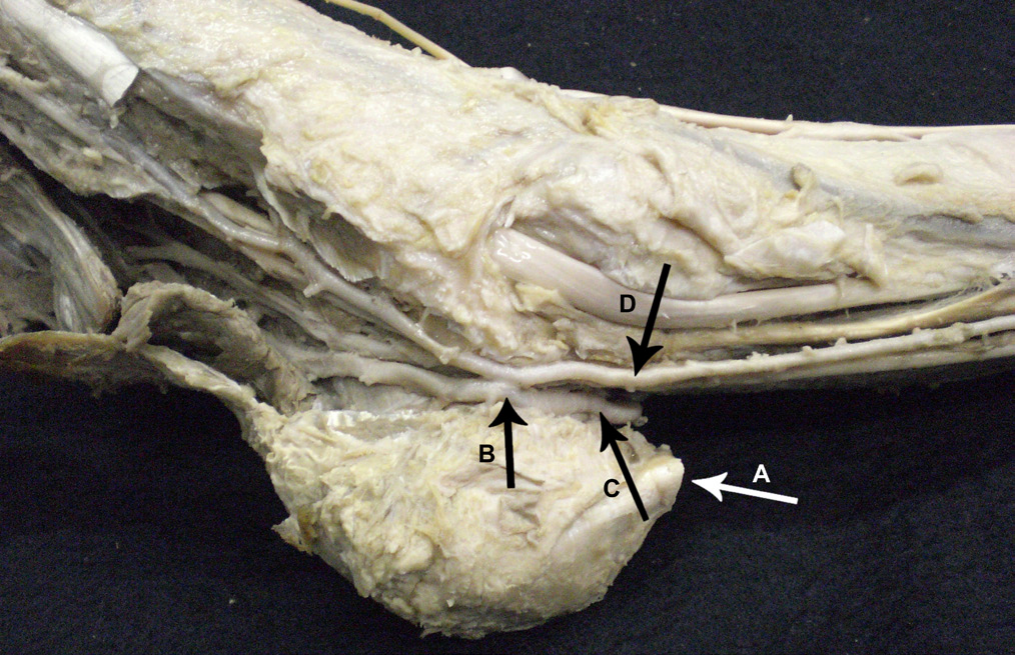
Medial view of the retro-maleolar region of the right ankle. The FA (C) and PTA (D) are visible following their courses, the anastomosis in “X” (B) can be observed, and the triceps surae tendon (A) is visible.

In this case, the origins of both arteries were as normal, but in the ankle the vessels underwent an “X-shaped” anastomosis, prior to the origin of the plantar branches from the PTA (Figure 2).

## DISCUSSION

Studies showed that the arteries of the lower limb are derived from the dorsal region of the umbilical artery, which forms the sciatic artery (SA), a branch of the internal iliac artery.^4^^,^ 9 The SA is the main supplier of blood to the early lower limb bud. During stage 16 (14 mm embryo), the femoral artery appears as a branch of the external iliac artery and forms an anastomosis with the SA, thereby becoming the source of the main blood supply to the lower limb.^9^^,^ 10 Afterwards, the proximal portion of the SA regresses and is represented by the inferior gluteal artery and the sciatic nerve artery. Its middle and distal portions persist to form the popliteal and fibular arteries. A superficial popliteal artery that passes superficially to the popliteus muscle gives origin to the ATA. The PTA is formed by an anastomosis between the early distal portions of the femoral artery and the popliteal artery. Its regular anatomy is completed by 3 months of gestation.^9^^,^ 10 ^,^ 17 ^,^ 18

Anatomic variants of these vessels often appear as result of persistent primitive arterial segments, segmental hypoplasia, abnormal fusions, or complete absence; furthermore, these mechanisms can frequently occur in combination.^4^^,^ 10 ^,^ 17 ^,^ 18 Hypoplasia of one of these vessels can cause abnormal blood supply to the foot.^19^^,^ 20

Variations in the origin and course of the PTA and the FA have often been described after being detected either during dissection of cadaveric specimens or by arteriograms, Doppler exams, and duplex scanning of living subjects. In our analysis, we found that the PTA can be absent, hypoplastic or replaced in its distal portion or replaced altogether by the FA^1^^,^ 4 ^,^ 6 ^,^ 19; it can penetrate the interosseous membrane to join the ATA;^1^ it may turn anteriorly and replace the ATA;^1^ it may supply all the common digital arteries^1^; it can arise proximally to the ATA;^21^ it can arise at the knee joint, above the tibial plateau;^2^^,^ 4 ^,^ 19 ^,^ 21 it can arise from a common trunk with the ATA;^19^ and it can arise directly from the popliteal artery.^4^ Additionally, the FA can be hyperplasic, hypoplastic, absent or replaced by the PTA;^1^ it can sometimes give rise to the ATA;^1^ it can arise from the ATA;^10^ it can become the peronea magna artery in cases of absence of ATA and PTA;^10^^,^ 22 it may give origin to the dorsalis pedis artery;^2^ and it may arise directly from the popliteal artery.^12^ Our report is the first one to present this unusual form of anastomosis between the PTA and the FA, although both arteries had regular trajectories thereafter.

In cases of hypoplasia or aplasia of the PTA, the FA exhibits compensatory hyperplasia, giving off branches to what would normally be the PTA’s vascular territory.^2^^,^ 4 ^,^ 7 In the foot, if the PTA is absent, the FA gives rise to the lateral plantar artery, while the medial plantar artery is usually absent.^6^^,^ 7 Cases have been described in the literature of idiopathic clubfoot associated with absence of the PTA.^6^ There are also cases of aplasia of both the PTA and the ATA and in these cases the FA is responsible for the blood supply to the whole leg.^2^^,^ 4

The FA also provides many fasciocutaneous and musculocutaneous perforators to supply the skin and muscles, albeit they are more common in the distal portion of the FA. These branches exhibit arterial anastomoses with each other in the subcutaneous tissue, and it appears that the second, third and fourth musculocutaneous branches have larger diameters and should be used for fibular flaps.^11^^,^ 12

Knowledge of these variant patterns is important for evaluation of lower limb arteriograms and also in respect for their clinical and surgical significance for procedures such as vascular grafts, surgical repair, transluminal angioplasty, and embolectomy and for diagnosis of arterial injury.^4^^,^ 11 ^,^ 12 ^,^ 19 ^,^ 21 ^-^ 24 In diabetics, chronic arteriopathy is very dangerous, because it causes disruption of the distal perfusion after many years of progression. This can lead to insufficient blood flow, causing ischemia and subsequent necrosis, thus requiring amputation, although this situation is changing due to recanalization techniques.^16^

Although rare, true aneurysms of the PTA have been described in the literature and they can often compromise vessels and nerves.^15^ Pseudoaneurysms of the PTA are also uncommon, but it has been reported that they can cause nonunion of tibial fractures and symptoms of lower limb ischemia due to thrombosis and distal embolization.^14^

The free fibular flap (FFF) has recently come to be preferred for correction of mandible defects because of its low morbidity and the fact that this procedure allows a two-team approach.^12^ Recent studies have also proven that the fibular artery perforator flap is useful for reconstruction of the skull, and its main advantage would be the mobility of each component of the tissue.^24^ However, in cases of aplasia or hypoplasia of the PTA, preoperative Doppler, ultrasound, MRI or angiogram may be needed after an FFF procedure to avoid lower leg ischemia, since the FA would have been providing the blood supply to the PTA’s territory.^12^

Since the PTA and the FA provide the blood supply to the foot, their distal portions and trajectories should be studied, due to the fact that ankle arthroscopy is becoming a popular procedure to treat arthritis and anatomical variations on this region can increase risk.^10^^,^ 20 Distal branching patterns - such as ours - are often difficult to assess particularly in the setting of peripheral vascular disease and artery reconstruction.^2^^,^ 19 An occlusion of the anastomosis described here could cause full ischemia of the foot, since both arteries are involved in supplying blood to this region.

Traumatic events in the Achilles region require free-tissue transfer. A study performed by Vaienti et al.^13^ reported that flaps using these vessels are safe and their perforators enable surgeons to extract more tissue to use in the reconstruction of the Achilles region. Furthermore, recent studies have reported that flaps from the lateral calcaneal artery (which is normally a branch of the FA, although it can sometimes arise from the PTA) is a good option for small injuries in the calcaneal region, but this is contraindicated in cases in which the lateral calcaneal artery provides the supply for the whole foot.^25^ In the present case, we would advise not to use the distal portion of the PTA or the FA for vascular flaps, since any type of iatrogenic injury during surgery could cause massive bleeding and hemostasis would be difficult, leading to limb ischemia or death because of the anastomosis in an unusual pattern.

Any type of vascular surgery in this area should be planned preoperatively, since there are many variations of the vessels in this region and some patterns of vascular distribution can contraindicate fibular flaps. Knowledge about the lower limb arteries is extremely important in a number of conditions, since anatomic variations can cause difficulties with diagnostic and surgical procedures.

## References

[1] Bergman R, Thompson S, Afifi A, Saadeh F (1988). Compendium of human anatomic variation: text, atlas, and world literature.

[2] Kim D, Orron D, Skillman J (1989). Surgical significance of popliteal arterial variants: a unified angiographic classification. Ann Surg.

[3] Hollinshead W (1976). Functional anatomy of the limbs and back.

[4] Kil S, Jung G (2009). Anatomical variations of the popliteal artery and its tibial branches: analysis in 1242 extremities. Cardiovasc Intervent Radiol.

[5] Abou-Foul A, Borumandi F (2016). Anatomical variants of lower limb vasculature and implications for free fibula flap: systematic review and critical analysis. Microsurgery.

[6] Karaolanis G, Galyfos G, Karanikola E, Palla V, Filis K (2015). Absence of clinical and hemodynamic consequences due to posterior tibial artery congenital aplasia. Case Rep Vasc Med.

[7] Zheng M, Chen C, Qiu Q, Wu C (2016). Ultrasound in the diagnosis of anatomical variation of anterior and posterior tibial arteries. Med Ultrason.

[8] Testut L, Jacob O (1944). Tratado de anatomía topográfica com aplicaciones médico-quirúrgicas.

[9] Goss CM (1973). Gray's anatomy of human body.

[10] Mauro M, Jaques P, Moore M (1988). The popliteal artery and its branches: embryologic basis of normal and variant anatomy. AJR Am J Roentgenol.

[11] Saitoh S, Hata Y, Murakami N (2001). The ‘superficial’ peroneal artery: a variation in cutaneous branching from the peroneal artery, nourishing the distal third of the leg. Br J Plast Surg.

[12] Choi S, Kim H, Koh K, Chung I, Cha I (2001). Topographical anatomy of the fibula and peroneal artery in Koreans. Int J Oral Maxillofac Surg.

[13] Vaienti L, Calori G, Leone F, Brioschi M, Parodi P, Marchesi A (2014). Posterior tibial artery perforator flaps for coverage of Achilles region defects. Injury.

[14] Kalyan JP, Kordzadeh A, Hanif MA, Griffiths M, Lyall H, Prionidis I (2015). Nonunion of the tibial fracture as a consequence of posterior tibial artery pseudoaneurysm. J Surg Case Rep.

[15] Sagar J, Button M (2014). Posterior tibial artery aneurysm: a case report with review of literature. BMC Surg.

[16] Pernès J, Auguste M, Borie H (2015). Infrapopliteal arterial recanalization: a true advance for limb salvage in diabetics. Diagn Interv Imaging.

[17] Arey L (1974). Developmental anatomy.

[18] Lippert H, Pabst R (1985). Arterial variations in man: classification and frequency.

[19] Day C, Orme R (2006). Popliteal artery branching patterns: an angiographic study. Clin Radiol.

[20] Vazquez T, Rodríguez-Niedenfuhr M, Parkin I, Viejo F, Sauno J (2006). Anatomic study of blood supply of the dorsum of the foot and ankle. Arthroscopy.

[21] Sanders R, Alston G (1986). Variations and anomalies of the popliteal and tibial arteries. Am J Surg.

[22] Betz LH, Betz BW (2009). Peronea arteria magna. Pediatr Radiol.

[23] Ozgur Z, Ucerler H, Ikiz ZAA (2009). Branching patterns of the popliteal artery and its clinical importance. Surg Radiol Anat.

[24] Heredero S, Solivera J, García B, Dean A (2016). Osteomyocutaneous peroneal artery perforator flap for reconstruction of the skull base. Br J Oral Maxillofac Surg.

[25] Burusapat C, Tanthanatip P, Kuhaphensaeng P, Ruamthanthong A, Pitiseree A, Suwantemee C (2015). Lateral calcaneal artery flaps in atherosclerosis: cadaveric study, vascular assessment and clinical applications. Plast Reconstr Surg Glob Open.

